# Cellular senescence: a double-edged sword in cancer therapy

**DOI:** 10.3389/fonc.2023.1189015

**Published:** 2023-09-12

**Authors:** Shuai Xiao, Dongmin Qin, Xueyang Hou, Lingli Tian, Yeping Yu, Rui Zhang, Hao Lyu, Dong Guo, Xing-Zhen Chen, Cefan Zhou, Jingfeng Tang

**Affiliations:** ^1^ National “111” Center for Cellular Regulation and Molecular Pharmaceutics, Key Laboratory of Fermentation Engineering (Ministry of Education), Hubei University of Technology, Wuhan, China; ^2^ Cooperative Innovation Center of Industrial Fermentation (Ministry of Education & Hubei Province), Hubei Key Laboratory of Industrial Microbiology, Hubei University of Technology, Wuhan, China; ^3^ Membrane Protein Disease Research Group, Department of Physiology, Faculty of Medicine and Dentistry, University of Alberta, Edmonton, AB, Canada

**Keywords:** cellular senescence, cell cycle arrest, SASP, cancer therapy, senotherapy

## Abstract

Over the past few decades, cellular senescence has been identified in cancer patients undergoing chemotherapy and radiotherapy. Senescent cells are generally characterized by permanent cell cycle arrest as a response to endogenous and exogenous stresses. In addition to exiting the cell cycle process, cellular senescence also triggers profound phenotypic changes such as senescence-associated secretory phenotype (SASP), autophagy modulation, or metabolic reprograming. Consequently, cellular senescence is often considered as a tumor-suppressive mechanism that permanently arrests cells at risk of malignant transformation. However, accumulating evidence shows that therapy-induced senescence can promote epithelial-mesenchymal transition and tumorigenesis in neighboring cells, as well as re-entry into the cell cycle and activation of cancer stem cells, thereby promoting cancer cell survival. Therefore, it is particularly important to rapidly eliminate therapy-induced senescent cells in patients with cancer. Here we review the hallmarks of cellular senescence and the relationship between cellular senescence and cancer. We also discuss several pathways to induce senescence in tumor therapy, as well as strategies to eliminate senescent cells after cancer treatment. We believe that exploiting the intersection between cellular senescence and tumor cells is an important means to defeat tumors.

## Cellular senescence

**Figure 1:**
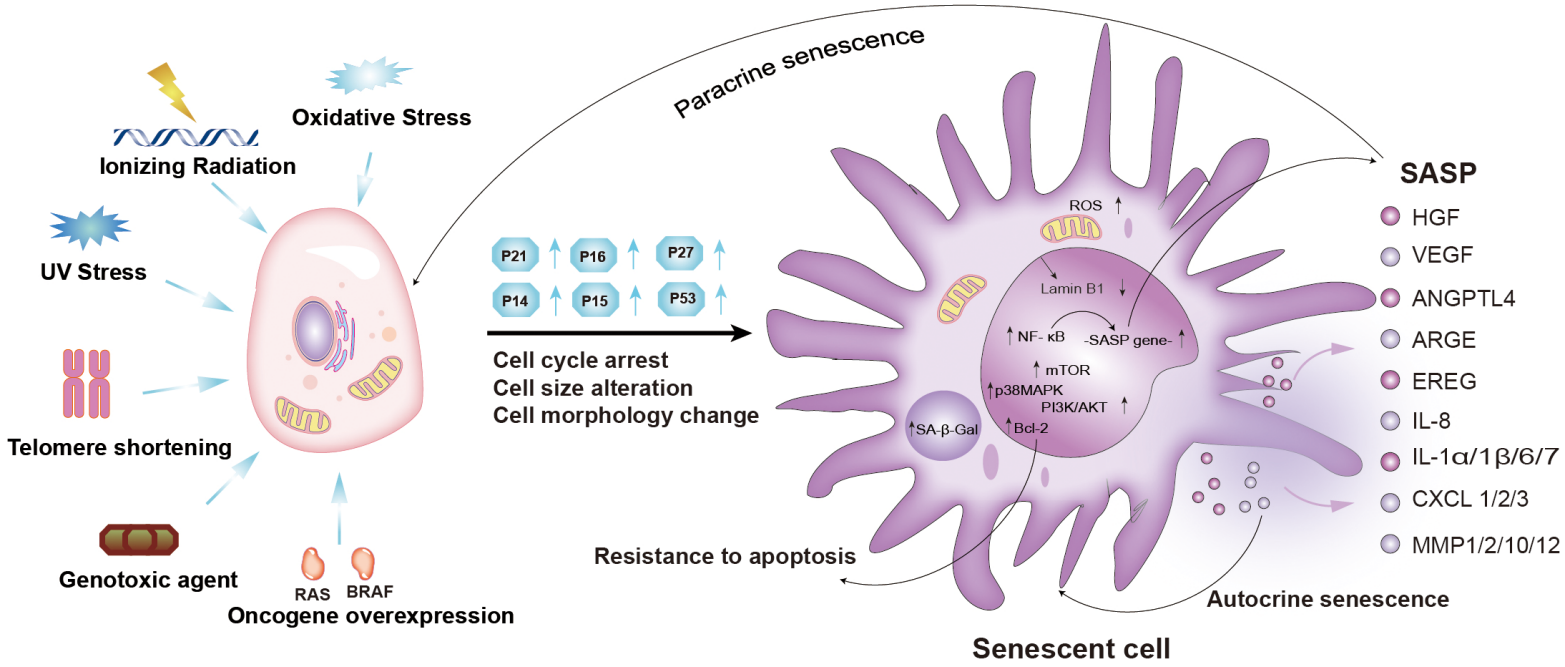
Mechanisms and characteristics of senescent cells, and components of the senescence-induced secretory phenotype (SASP). Cellular senescence can be induced by oxidative stress, ionizing radiation, UV stress, telomere shortening, genotoxic agents, and oncogene overexpression. Senescent cells exhibit permanent cell cycle arrest, increased cell cycle inhibitors (p21, p16, p27), and morphological and molecular alterations. Cellular senescence is reinforced and spreads in an autocrine or paracrine manner. PI3K-AKT-mTOR, p38 MAPK, NF-κB, cGAS-STING, TGF-β, and JAK-STAT signaling pathways were activated (2–6). (Ras, RAS Proto-Oncogene; BRAF, B-Raf Proto-Oncogene; SASP, senescence-associated secretory phenotype; NF-κB, nuclear factor-kappa B; mTOR, mammalian target of rapamycin; BCL-2, B-cell lymphoma-2; MAPK, mitogen-activated protein kinase; PI3K, phosphatidylinositol-3-kinase; IL, interleukin; HGF, hepatocyte growth factor; VEGF, vascular endothelial growth factor; ANGPTL4, angiopoietin-like protein 4; ARGE, Amphiregulin; EREG, Epiregulin; IL8, interleukin 8; IL1α/1β/6/7, interleukin 1α/1β/6/7; CXCL1/2/3, C-X-C chemokine ligand 1/2/3; MMP1/2/10/12, matrix metalloproteinases1/2/10/12; ROS, Reactive oxygen species).

**Figure 2:**
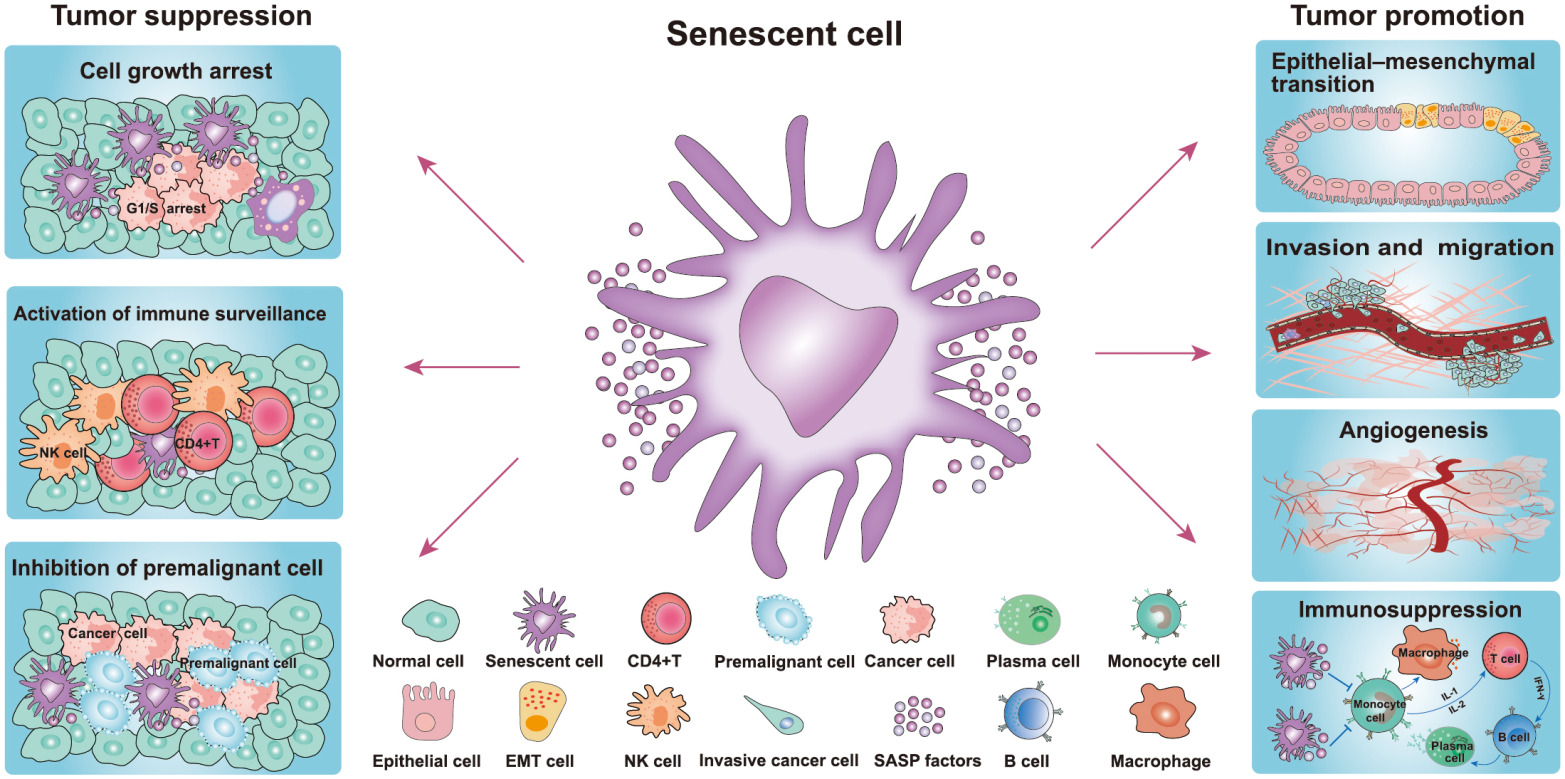
Role of senescence in cancer. Senescent cells play a dual role in the occurrence and development of tumors. The pro-tumorigenic effects of senescent cells: senescent cells include their ability to promote cancer cell proliferation, migration, invasiveness, angiogenesis, and EMT, while the antitumor effects include inhibition of the growth of premalignant cells and cancer cells and activation of immune surveillance (20, 21). (NK cell, natural killer cell; EMT, epithelial-mesenchymal transition cell).

Cellular senescence is a cell state characterized by permanent cell cycle arrest, elevated senescence-associated β galactosidase (SA-β-gal) activity, resistance to apoptotic stimuli, and deregulated metabolism (1). Compared to the morphology and molecular biology of proliferating cells, those of senescent cells undergo remarkable changes (**Figure 1**) (7). Senescent cells become flattened and the nucleus becomes larger, representing important hallmarks of the senescent state (7). The altered morphology of senescent cells may be due to protein accumulation caused by decreased proteasome peptidase activity in senescent cells (8). SA-β-gal, a lysosomal enzyme, is another important hallmark of senescent cells (9, 10). Indeed, SA-β-gal was reported as the first marker used to identify cellular senescence given that its activity is significantly increased in almost all senescent cells (10). SA-β-gal activity has also been used as a specific marker of cancer cell senescence (10). However, the lower SA-β-gal has been detected in some non-senescent cells, such as macrophages, hair follicles, and sebaceous glands (11–13). Despite this, SA-β-gal activity remains the most widely used marker of cellular senescence. Persistent cell cycle arrest is another hallmark feature of senescence (14). Cell cycle arrest is dependent on cyclin-dependent kinase (CDK) inhibitors, such as p16 INK4a(CDKN2A), p27Kip1 (CDKN1B), and p21Waf1 (CDKN1A, CIPI) (**Figure 1**) (14). The p53/p21WAF1/Cip1 pathway and p16 Ink4a/retinoblastoma (Rb) pathways are often activated in senescent cells, causing increased expression of p16 Ink4a, p27 kip1, and p21WAF1/Cip1 (**Figure 1**) (15–17). The accumulation of p16 Ink4a and p21WAF1/Cip1 inhibits CDK2 and CDK4/6 activity, thereby decreasing the phosphorylation of Rb (p-Rb) (17). It is well-established that Rb binds to the E2F family at low phosphorylation levels, inhibiting the activity of E2F family transcription factors and causing cell cycle arrest (12). Therefore, the level of p-Rb is another important marker of cellular senescence (12). Senescent cells secrete various different cell factors, including growth modulators, angiogenic factors, pro-inflammatory cytokines and chemokines, matrix metalloproteinases, and other proteases, which collectively constitute a senescence-associated secretory phenotype (SASP) (18). The SASP is the most significant marker of cellular senescence caused by persistent DNA damage response (DDR) (19). The development of a SASP involves a relatively complex regulatory network, which may involve PI3K-AKT-mTOR signaling, nuclear factor kappa-B (NF-κB) signaling, the p38 MAPK pathway, the cGAS-STING pathway, inflammasome activation, transforming growth factor-β (TGF-β) signaling, and anus kinase/signal transduction and activator of transcription (JAK-STAT) signaling (**Figure 1**) (2–6). Numerous studies have shown that the development of a SASP in senescent cells is a double-edged sword (**Figure 2**) (22–24). Although SASP can eliminate senescent cells or cancer cells by activating immune responses (22), persistent SASP signaling may promote tumorigenesis in neighboring normal cells, as well as epithelial-mesenchymal transition (23, 24). Moreover, the downregulation of the nuclear lamina protein laminB1 and the increased expression of p19ARF, γ-H2AX, and PAI-1 in senescent cells can also be used as biomarkers of cellular senescence (25). Therefore, it is necessary to detect multiple indicators to determine whether cellular senescence occurs.

## Types and roles of cellular senescence

Hayflick and colleagues first discovered the phenomenon of cellular senescence by the finding that normal diploid fibroblasts in culture entered a permanent cell cycle after a limited number of cell divisions (26). Several decades later, Harley observed that DNA replication leads to telomere damage and then induces cellular senescence, which is now known as replicative senescence (27). Subsequently, another study demonstrated that the overexpression of oncogenes induced senescence in primary cells, which was termed oncogene-induced senescence (OIS) (28). As is well-known, oncogenes play a key role in stimulating cell proliferation and tumorigenesis in many cancer types (28). For this reason, OIS works as a tumor-suppressive mechanism to prevent aberrant proliferation following an oncogenic stimulus. A previous study demonstrated that hyper-expression of the *Ras* gene in primary lung fibroblasts (IMR90) induced cellular senescence (11). Since then, an increasing number of oncogenes (e.g., AKT, PTEN, and E2F1) have been found to induce cellular senescence (28). Moreover, it is increasingly recognized that cellular senescence can be induced by several types of stress signals, including chemotherapy, irradiation, cytokine treatment, oxidative stress, and induced pluripotent stem (iPS) cell reprogramming (**Figure 1**) (28). Among them, cellular senescence caused by DNA double-strand breaks (DSB) in cancer cells and paracancerous cells is most common after cancer treatment, which is known as therapy-induced senescence (TIS) (29). Although senescence could conceivable be used as a tumor suppressor mechanism to prevent the growth of cancer cells due to its cell cycle arrest property, there is also substantial evidence to suggest that the persistence of senescent cells leads to local or systemic toxicity organ dysfunction and inflammation, which may promote the occurrence and development of cancer (23, 24). Thus, cellular senescence has a dual effect on cancer, suggesting that the benefits and drawbacks of cellular senescence should be cautiously considered.

## Advantage of cellular senescence: inhibition of tumor progression

Immortal replication and uncontrolled proliferation, which cause cancer cell uncontrolled growth, are important hallmarks of cancer cells (30, 31). In contrast, it is well established that senescent cells are associated with cell cycle arrest and inhibition of cell proliferation (14). Hence, inducing cellular senescence of cancer cells is considered a potentially effective strategy against cancer (32). Senescent cells are in a stable state of cell cycle arrest and are considered as a natural barrier to tumorigenesis. Mechanistically, increased expression of the cell cycle suppressor CDKI leads to cell cycle arrest in the G1 or G2/M phase (20, 21, 33). Due to cell cycle arrest and mitotic inhibition, premalignant cells will not become cancerous, even after treatment-induced damage and stress (34). Similarly, senescent cancer cells also undergo cell cycle arrest and proliferation inhibition, thereby inducing tumor growth inhibition (**Figure 1**) (34). Additionally, an increasing body of evidence has indicated that OIS, which is driven by activated oncogenes such as Ras and cyclin E, can effectively prevent excessive proliferation of transformed cells, thereby inhibiting oncogenesis in the early stages (**Figure 1**) (28, 35). Once the OIS mechanism is inactivated, premalignant cells will bypass senescence and transform into aggressive malignant cells (35). Indeed, the tumor-suppressive role of OIS is supported by many mouse studies. Senescence-related genes play an important role in the process of tumor suppression, while the disruption of senescence-essential genes predisposes organisms to develop cancer (36). A previous animal study demonstrated that mice develop cancer at an early age due to inactivation of p53 or p16 proteins (37).

The SASP of senescent cells also inhibits the occurrence and development of cancer (**Figure 2**) (38). The components of the SASP can induce and enhance cell growth arrest by autocrine or paracrine mechanisms. Among the numerous SASP components, interleukin (IL-1, IL-6, and IL-8) play important roles in the SASP response in senescent cells and adjacent tissues (39). Indeed, it has been reported that IL-1 is the key factor responsible for activating and maintaining the SASP during cellular senescence (3, 40). The SASP also serves as the main medium for senescent cells to communicate with neighboring cells, which also plays an antitumor role. Additionally, senescent cells secrete multiple SASP factors through the paracrine pathway, including TGF-β family ligands, chemokine (C-C motif) ligand 2 (CCL2), chemokine (C-C motif) ligand 20 (CCL20), and vascular endothelial growth factor (VEGF) (**Figure 1**) (3). These factors damage the DNA of neighboring cells and increase the expression of p16, p21, and IL-8, thereby inducing senescence of adjacent cells (41). SASP can also activate the immune system to facilitate cancer cell clearance (41). It has been reported that premalignant senescent hepatocytes were cleared by SASP-mediated innate immune activation in mice via the secretion of several chemicals- and cytokines (**Figure 2**) (41, 42). Moreover, natural killer (NK) cells and M1 macrophages have also been shown to be recruited following the formation of the SASP to eliminate senescent cells or cancer cells (43). Taken together, the positive effects of cellular senescence are beneficial for the inhibition of tumor initiation and progression.

## Harmful effects of cellular senescence: Promotion of tumor progression

In addition to the aforementioned beneficial effects, there is also much evidence that senescent cells promote cancer development (**Figure 2**) (20, 21). These studies have indicated that senescent cells contribute to the proliferation, migration, invasiveness, angiogenesis, and epithelial-mesenchymal transition (EMT) of tumors (**Figure 2**) (21). Indeed, SASP plays a key role in tumor-promoting processes, such as angiogenesis, stemness, genotoxicity chronic inflammation, invasion and migration, and immunosuppression. Moreover, in the breast carcinoma mouse model, SASP factors have been found to mediate cancer relapse by stimulating the malignant transformation of surrounding non-senescent breast cancer cells (**Figure 2**) (43). Senescent cells persistently secrete various SASP factors through the paracrine pathway to affect adjacent tissues, which plays the most important role in pro-tumorigenesis (43). The SASP pro-tumorigenic effect was first discovered in the study that senescent fibroblasts co-cultured with preneoplastic epithelial cell lines (44). The results showed that senescent fibroblasts promoted the proliferation rate of preneoplastic epithelial cell lines, but failed to influence the proliferation rate of normal epithelial cells (44). The authors of this study further evidenced that senescent fibroblasts accelerate the development of epithelial tumors *in vivo* following co-injection of epithelial cancer cells and senescent fibroblasts under the skin of the mice (44). In another study, premalignant breast epithelial cells were found to acquire cancer cell properties when co-cultured with senescent fibroblasts (18). Furthermore, researchers found that senescent fibroblasts secrete MMP-3 and collagen thereby causing epithelial cells to occur undergo morphological and functional differentiation (19). In an animal xenograft study comprising five mice, a group of MDA231 cells generated tumors that reached 300–400 mm^3^ in two mice at day 45. However, the group of MDA231 cells co-injected with senescent fibroblasts generated tumors that reached 300–1600 mm^3^ in four of five mice (44). These results strongly suggested that senescent fibroblasts greatly stimulate or facilitate tumorigenesis.

The SASP also promotes the invasion of premalignant epithelial cells and the EMT of noninvasive cancer cells. It has been reported that the migration of breast cancer cells was significantly enhanced after co-cultured with senescent cells, with IL-6 and IL-8 found to play a key role in the invasiveness of breast cancer cells by blocking antibodies of IL-6 and IL-8 (19). In other studies, IL-6 and IL-8 were also proved to positively promote the EMT of cancer cells (45, 46). IL-6 and IL-8 activate STAT3 signaling, which in turn activates EMT-related genes and MMP gene expression (47, 48). MMPs positively degrade the extracellular matrix, which contributes to the acquisition of mitogens and growth factors (49). Accompanied by the reduction of β-catenin and E-cadherin expression, a mesenchymal phenotype was found in breast cancer cells following co-culture with senescent fibroblasts (19). Senescent cells can also promote the formation of blood vessels by secreting VEGF (50). As a component of the SASP, VEGF can promote angiogenesis and contribute to the malignant growth of cancer cells (5, 50). Therefore, SASP components can directly or indirectly promote cancer cell growth, invasion and metastasis, and tumor vascularization. Taken together, these results reveal that cellular senescence has a dual effect on cancer, suggesting that we need to be more cautious about the benefits and drawbacks of cellular senescence.

## Cancer therapy induces cellular senescence

**Figure 3:**
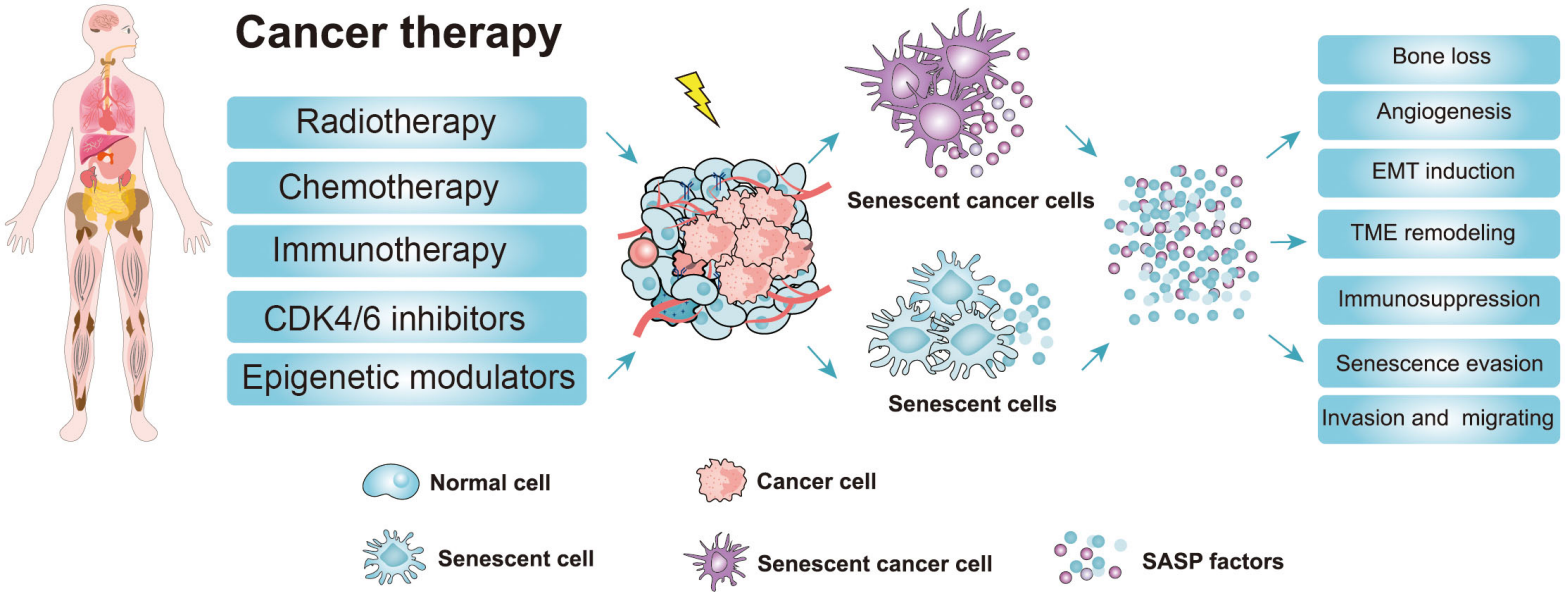
Cancer therapy strategies and the adverse effects of the senescence-induced secretory phenotype (SASP) factors secreted by therapy-induced senescent cells. Cancer therapy such as chemotherapy, radiotherapy, and targeted therapy can promote senescence in cancer cells and nonmalignant cells (51, 52). The accumulation of therapy-induced senescent cells is detrimental to the organism due to the non-cell autonomous effect of the SASP. The SASP can potentiate various aspects of tumorigenesis. Moreover, the SASP factors contribute to therapy resistance and the harmful effects of cancer therapy (CDK4/6 inhibitors, cyclin-dependent kinase 4/6 inhibitors; EMT induction, epithelial-mesenchymal transition induction; TME, Tumor microenvironment; SASP, senescence-induced secretory phenotype).

Chemotherapy and radiotherapy are the major treatment methods for patients with cancer, although many others, such as immunotherapy, epigenetic modulators, and CDK4/6 inhibitors, are also in use (**Figure 3**) (51, 52). After therapy, most cancer cells will undergo apoptosis or senescence, leading to the death of cancer cells. Senescence caused by tumor cells in response to chemotherapy and radiation is termed TIS (**Figure 3**) (52–54). As well as normal senescent cells, senescent cancer cell phenotypes, such as growth arrest, changed morphology, and elevated SA-β-gal activity, are universally present (51). However, the other senescent markers (p-Rb, p53, p-p53, p21, p16, γ-H2A, and LaminB1) do not always reflect the senescent state of cancer cells due to the heterogeneity of different tumors (51, 52). Therefore, it is important to comprehensively evaluate whether cancer cells become senescent after chemotherapy. Here, we focus on TIS induced by chemotherapy, radiotherapy, and CDK4/6 inhibitor therapy.

### Chemotherapy-induced cancer cellular senescence

Chemotherapy is the use of cytotoxic compounds, such as doxorubicin, etoposide, aphidicolin, bleomycin, cisplatin, mitoxantrone, retinol, and hydroxyurea, to cause unrepairable DNA damage to cancer cells and impair mitosis (52). Chemotherapeutic drugs are usually highly cytotoxic, with the ability to cause DNA damage and inhibit mitosis in tumors and surrounding normal cells. Numerous studies have demonstrated that those chemotherapeutic drugs cellular senescence of cancerous cells at the clinical concentration (53, 54). Cancer cells that have received chemotherapy have three possible fates, including senescence, apoptosis, and necrosis, which may be may be related to the dose and duration of treatment (12). Doxorubicin is the most common topoisomerase inhibitor, which can block topoisomerase enzymes from re-ligating DNA strands after supercoil unwinding, thereby preventing replication (55). Doxorubicin is used to treat various cancers and can induce the senescence of cancer cells, including lymphoma, breast, lung, and acute lymphocytic leukemia (55). One study showed that the 25 nM dosing of doxorubicin induces senescence in prostate cancer cells, while the 250 nM dosing of doxorubicin induces prostate cancer cell apoptosis (56). Additionally, etoposide and cisplatin have been reported to induce senescence of non-small cell lung cancer (NSCLC) H1299 cells (57).

### Radiotherapy-induced cancer cellular senescence

Radiotherapy is an effective cytotoxic therapy method for many cancer types, including skin, breast, head, bladder, lung, and prostate cancers (58). Ionizing radiation (IR) is a unique form of radiation that possesses high-energy electrically charged ions, which irreversibly damage DNA to induce cancer cell death (59). The exposure of normal or cancerous cells or tissues to IR can trigger a senescent response (59). Compared to chemotherapy, radiotherapy causes minor damage to noncancerous tissue areas because it targets the site of cancer rather than being administered systemically (60). Increasing evidence indicates that IR can also cause cellular senescence of cancerous cells (24, 61). Senescent markers, such as SA-β-gal, p16, p21, and SASP, have been identified in cancer cells after IR treatment (61). One study indicated that the occurrence of senescence following radiotherapy is heavily dependent on the patient’s p53 status (62). For example, when missense mutations occur in the p53 DNA-binding domain, breast cancer cells undergo apoptosis rather than senescence after IR treatment (62). In another study, the wild-type p53 of glioblastoma multiforme cells was found to accelerate the senescent response to IR treatment. However, the p53 mutation of glioblastoma multiforme cells regenerates their ability to proliferate when treated with IR (63). Moreover, IR also can induce normal cellular senescence *in vitro* and *in vivo* (59, 60). Previous studies have indicated that the senescent markers are significantly enhanced after IR treatment in normal cells (59–61). Additionally, when normal mice were treated with sublethal doses of IR have been shown to develop progressive premature frailty (64). Therefore, the major direction for future advances in radiotherapy will be to balance the dose of radiotherapy, in favor of the host by modulating the therapeutic approach, mitigating the unwanted effects, and protecting normal tissue from radiation damage.

### CDK4/6 inhibitors induced cancer cellular senescence

It is well established that CDK4/6 plays a key role in the cell cycle transition (from G1 to S phase) (65). Therefore, CDK4/6 as an important antitumor target, was developed as a chemotherapeutic drug for cancer therapy (66, 67). At present, several CDK4/6 inhibitors have been approved and have been applied in clinics, including palbociclib, ribociclib, and abemaciclib. Palbociclib was the first CDK4/6 inhibitor to be approved for patients with estrogen receptor-positive (ER+) advanced breast cancer (68). Preclinical models of breast cancer research showed that palbociclib induces breast cancer senescence and reduces the tumor volume by activating immunosurveillance (69). Palbociclib has also been reported to induce cellular senescence in melanoma, liposarcoma, gastric cancer, and hepatocellular carcinoma, thereby inhibiting further growth of these cancers (67, 70). Further research showed that palbociclib induces senescence of melanoma cells by inhibiting mammalian target of rapamycin (mTOR) signaling (70). However, it has also been reported that the senescence induced by palbociclib is not always stable, which may be related to the fact that palbociclib does not damage DNA when used to treat cancer cells (71, 72). The duration of treatment with palbociclib is another important factor in the induction of cellular senescence, in that long-term treatment with palbociclib promotes cell cycle arrest in both normal and cancer cells (72, 73). Additionally, the variety of cancer cell types also is an important reason for the different sensitivity to palbociclib. For example, palbociclib has been shown to induce irreversible cell cycle inhibition and senescence in MCF7 cells, but reversible cycle inhibition in bone cancer cells (74). Furthermore, palbociclib induces p53-deficient melanoma cells G1 arrest with prolonged treatment, which indicates that palbociclib-induced senescence is independent of the p53 pathway (70). In addition to palbociclib, ribociclib and abemaciclib were subsequently developed and approved by the FDA for the treatment of patients with hormone receptor-positive (HR+) human epidermal growth factor receptor 2-negative (HER2−) breast cancer (67). Compared to palbociclib, abemaciclib induces cancer cell cycle inhibition and senescence that is highly dependent on the p53 pathway (75). In view of the above adverse factors, the antitumor effect of CDK4/6 inhibitors needs to be further investigated.

Taken together, these findings show that cancer therapy induces cellular senescence of cancerous cells, leading to cancer cell death or cell growth arrest. Especially for those apoptosis-resistance cancer cells, TIS seems to be a promising way to inhibit cancer cell proliferation (76). Nonetheless, the harmful effects caused by TIS should also be considered. For example, some non-cancer cells are also induced into senescence during tumor therapy, leading to substantial side effects and the progression of chronic diseases (77). Senescent cells secrete a variety of SASPs, which can not only activate the immune system to inhibit tumor growth but also promote EMT and angiogenesis to enhance tumor migration and metabolism (18, 50). Additionally, persistent SASP signals can also increase the likelihood of treatment resistance, thereby affecting the therapeutic effect which is conducive to the development of cancer (78). Accumulating evidence reveals that the removal of therapy-induced senescent cells can eliminate the detrimental outcomes of senescent cells, which significantly improved the therapeutic effect and thus extended the life span of experimental animals (24, 79). Therefore, selective removal of senescent cells after cancer treatment may be a promising therapeutic approach.

## “One-two punch” therapy: a novel anticancer strategy

**Figure 4:**
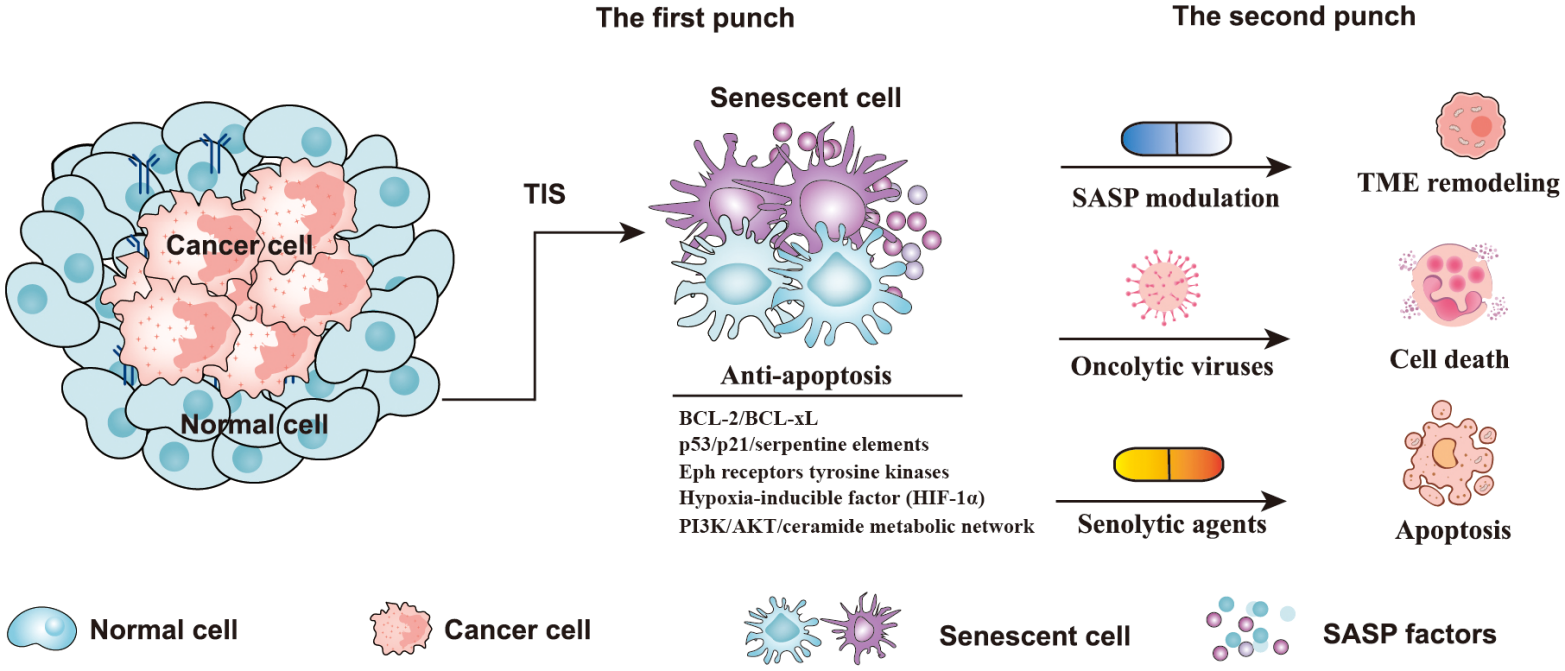
Strategies of a “one-two punch” for senotherapy-mediated cancer treatment. Senescent cells contain five anti-apoptotic pathways, including p53/p21/serpentine elements, the PI3K/AKT/ceramide metabolic network, Eph receptor tyrosine kinases, BCL-2/BCL-xL, and the hypoxia-inducible factor (HIF-1α) pathway (84). In the “one-two punch” approach for the treatment of cancer, the first punch: using a drug to induce senescence in cancer cells, and the second punch: using a second drug to kill senescent cancer cells. (TIS, therapy-induced senescence; TME, Tumor microenvironment; BCL-2, B-cell lymphoma-2; BCL-xL, B Cell Lymphoma/Leukemia × Long Form).

Recently, a “novel one-punch” approach was employed to treat patients with cancer and target TIS. As is well known, chemotherapy or radiotherapy kills cancer cells but also leads to the senescence of both cancer cells and normal cells (80). Despite the fact that cellular senescence can also effectively suppress tumor growth, the persistence of senescent cells after cancer treatment poses tumor-promoting risks, such as promoting cancer stemness, re-entering the cell cycle, and eventually contributing to tumor relapse (20, 57). The above phenomena have aroused the interest of researchers in eliminating senescent cells after cancer treatment to solve this problem (24, 81). Several preclinical and clinical studies have shown that the combination of targeted elimination of senescent cells with currently used cancer therapies not only mitigated the potential adverse effects of cancer therapy but also significantly reduced the risk of cancer progression (82, 83). Furthermore, removing senescent cells after cancer therapy can effectively alleviate the harmful effects, including cancer recurrence, cardiac dysfunction, and reduced bone marrow suppression (83). Therefore, the “one-two punch” strategy, based on sequential antitumor and antisenescence interventions, may represent an effective strategy to treat cancer (**Figure 4**). The aim of “one-two punch” therapies is to exploit the vulnerabilities of senescent tumor cells to eliminate tumors by senolytic agents. The timely elimination of senescent cells seems to be particularly important after antitumor therapy. Here we discuss several strategies that can be used to remove senescent cells, including senolytic agents, modulating SASP factors, and oncolytic viruses (**Figure 4**).

It has been reported that senescent cells have a high level of anti-apoptotic activity, which inhibits cell death (86). Senolytics agents have been reported to induce apoptosis of senescent cells by targeting anti-apoptotic pathways (87, 88). According to reports, senescent cells contain five anti-apoptotic pathways, including p53/p21/serpentine elements, the PI3K/AKT/ceramide metabolic network, Eph receptor tyrosine kinases, BCL-2/BCL-xL, and the hypoxia-inducible factor (HIF-1α) pathway (**Figure 4**) (84). To target these anti-apoptotic pathways, multitudinous natural or synthetic compounds were developed, such as BCL-2 family inhibitors, dasatinib and quercetin, heat shock protein 90 (HSP90) inhibitors, and others, such as fisetin, cardiac glycosides, and histone deacetylase (HDAC) inhibitors (84, 88). Quercetin is a natural flavanol that promotes the apoptosis of cells by inhibiting the PI3K signaling pathway (89). Quercetin has been proven to be more effective against senescent human endothelial cells (89). Dasatinib is an orally administered small molecule that has been approved for treating blood malignancies (90). Previous studies have shown that dasatinib can inhibit multiple tyrosine kinases, SRC family kinases, and platelet-derived growth factor receptors, and promote apoptosis of senescent cells by interfering with the EFNB signaling pathway (89, 90). Additionally, another study indicated that dasatinib is very effective against senescent human fat cells (89). The study found that dasatinib and quercetin preferentially induce apoptosis of senescent cells but not of non-senescent cells, which greatly enhances the potential of these two drugs as antitumor agents. Interestingly, the combination of dasatinib and quercetin (D+Q) showed a more effective killing of senescent cells than each agent alone (89). According to the report, the senolytic cocktail of D+Q was effective in reducing injury idiopathic pulmonary fibrosis (IPF) and against senescent alveolar epithelial cell types by selectively eliminating senescent cells (91, 92).

It is well known that BCL-2 family anti-apoptotic proteins not only protect against cell death by apoptosis but they also allow senescent cells to survive (93). Therefore, many antisenescence drugs have been developed to target these antiapoptotic proteins. Navitoclax (ABT263), a BH3 family mimetic, is a well-known orally administered small molecule that can inhibit the function of anti-apoptotic proteins, including BCL-2, BCL-w, and BCL-xL (79, 88). ABT-263 has been reported to interfere with senescence-associated anti-apoptosis, thereby selectively inducing senescent cell death *in vivo* (79). Indeed, ABT-263 can effectively eliminate senescent ovarian and breast cancer cells induced by the PARP inhibitor olaparib (85). Additionally, ABT-263 has been reported to interfere with Bcl-xL function in senescent melanoma and lung cancer cells, thereby promoting senescent cell death (94). However, the side effects of ABT263, such as the risks of thrombocytopenia and neutropenia, should be considered even though ABT263 promotes senescent cancer cell death and enhances the antitumor effect. Obatoclax is another pan-BCL2 family inhibitor that can induce cell apoptosis by inhibiting the activity of antiapoptotic proteins such as BCL-2, BCL-xL, and MCL-1 (95). In another study, obatoclax combined with the inhibitors of bromodomain and extra terminal (BETI) effectively promoted apoptosis of triple-negative breast cancer (TNBC) cells (83).

Usually, the senescent cells secrete a variety of cytokines and chemokines, which play an important role in maintaining the state of the senescent cells and contribute to tumor relapse (19). Therefore, SASP modulation may be an effective method for reducing senescent cells. For example, mTOR is an important regulator of the SASP, and the mTOR inhibitor temsirolimus promotes senescent cancer cell death and suppresses prostate and breast cancer cells (5, 96). Additionally, inhibiting STAT3 signaling reduces the side effects of SASP and suppresses prostate cancer cells (23). A previous study using a lymphoma mouse model indicated that the suppression of NF-kB signaling reduces SASP secretion, meanwhile, leading to tumor relapse and therapy resistance (97). Additionally, inhibiting mTORC1 with rapamycin reduces the translation of p53 and promoted prostate tumorigenesis in mice (98). Therefore, the efficiency and risk of the strategies employed to modulate SASP by inhibiting the pro-SASP signaling pathway need further investigation. Another method is to selectively inhibit a component of SASP, which may have a better function than the former due to less target impact. IL-1a is an important SASP initiator and IL-6/IL-8 are the most investigated pro-inflammatory SASP factors (5, 99). It has been reported that SASP tends to be activated in therapy-induced, oncogene-induced, and age-related senescent cells (100, 101). Many drugs targeting SASP factors have been developed and applied in clinical practice, including anakinra (IL-1 receptor drug), tocilizumab (IL-6 receptor inhibitor), and siltuximab (IL-6 antibody) (102–104).

Oncolytic viruses are a new class of therapeutic agents used for cancer treatment, which function by promoting antitumor responses that selectively replicate within tumor cells (105, 106). Although the ability of viruses to kill cancer cells has long been recognized, they have only recently been developed into antitumor drugs for clinical application in the past decade (106, 107). Oncolytic viruses also appear to be an effective strategy for inducing senescent cell death (107). Indeed, measles vaccine virus (MeV) was proven to be more susceptible to senescent cells than normal cells, thereby selectively killing senescent cancer cells (107). Therefore, oncolytic viruses are promising therapeutic agents for removing senescent cells after cancer treatment. Taken together, these findings highlight the existence of several suitable approaches to eliminate senescent cells combined with cancer therapy to reduce cancer relapse and improve the therapeutic effect.

## Discussion

Cellular senescence is a very complex physiological process involving multiple mechanisms. Although numerous studies have been conducted, the mechanisms underlying the effects of cellular senescence on tumors remain incompletely understood. Therefore, the pros and cons of therapy-induced cellular senescence are still controversial. It seems that cellular senescence is a double-edged sword in cancer, with senescent cells showing the ability to induce irreversible cell growth arrest and enhance innate immune surveillance, thereby inhibiting tumor development, as well as leading to the secretion of multiple SASP factors, which contribute to the proliferation, migration, invasiveness, angiogenesis, and EMT of tumors (20, 21). However, increasing evidence indicates that the beneficial effects of senescent cells in cancer are lower than the harmful effects. During cancer therapy, chemotherapy or radiotherapy kills the vast majority of cancer cells, while a few undergo senescence where they are unable to proliferate or die, which seems to favor inhibited growth. However, the senescent cancer cells remain in the tumor foci after treatment, and this persistence makes it difficult to eradicate the tumor and may lead to cancer recurrence and metastasis. Moreover, accumulating evidence suggests that SASP factors secreted by senescent cancer cells are critical to the recurrence and metastasis of the remaining cancer cells. Therefore, it remains challenging to make full use of the beneficial effects and overcome the deleterious effects of senescent cells in tumor therapy.

Based on previous results, we hypothesize that TIS-induced cellular senescence may have benefits for tumor therapy in the early stage, but that the harmful effects of senescent cells outweigh the benefits as senescent cells accumulate and SASP-associated factors continue to be released. If senescent cancer cells are removed in a timely manner after tumor therapy, the harmful effects of senescent cells seem to be eliminated. Moreover, inhibiting the production of specific SASP factors via the use of senolytics suppresses tumorigenesis. Following completion of anticancer therapy, senolytics prevent further tumorigenesis resulting from proinflammatory SASP factors. Thus, the “one-two punch” strategy may be an effective means to treat cancer. Compared to adjacent normal tissue cells, senescent cancer cells have great differences in metabolism (29). Hence, multiple senolytics are being developed to target the characteristics of senescent cells. Over the last decade of studies, these senolytics have been proven to have excellent performance in preclinical studies (108, 109). However, these agents have yet to be used in the clinic due to the selectivity of drugs to harmful senescent cells, systemic toxicities, and development of drug resistance. The “one-two punch” therapy strategy provides a potentially effective means for tumor therapy and enriches the methods of tumor therapy.

## Conclusions

Cellular senescence is a cell state characterized by permanent cell cycle arrest, which is induced by oncogenes, chemotherapy, radiotherapy, cytokine treatment, and oxidative stress. During tumor therapy, cellular senescence plays a dual role. Originally, cellular senescence is regarded as a key mechanism of tumor suppression due to the cell cycle arrest and proliferation inhibition characteristics of senescent cells. However, senescent cells also secrete large amounts of harmful SASP factors, which may induce cancer cell growth, invasion and metastasis, and tumor vascularization. With current knowledge, the harmful effects of senescent cells on cancer seem to outweigh the beneficial effects in some cases. Hence, the strategy of “one-two punch”, a combination of tumor therapy and senotherapy, will be crucial for tumor treatment and inhibition of tumor recurrence. Senotherapy, an effective method to remove senescent cells, is considered a potential strategy to suppress tumors. Developing effective pharmacologic methods to remove accumulated senescent cells or to impair the particular SASP factors secreted by senescent cells is the most important direction for cancer therapy in the future and also helps to balance the validity and potential risks of senotherapy.

## Author contributions

SX: Conceptualization, Software, Formal Analysis, Writing - Original Draft; DQ and XH: revised text and draft; LT and YY: revised text; RZ: Visualization, Investigation; HL: Software, Supervision; DG: Resources; X-ZC: Review & Editing; CZ and JT: Funding Acquisition, Resources, Supervision, Writing - Review & Editing. All authors contributed to the article and approved the submitted version.
